# Plasma Levels of Advanced Glycation Endproducts and Risk of Cardiovascular Events: Findings From 2 Prospective Cohorts

**DOI:** 10.1161/JAHA.121.024012

**Published:** 2022-07-29

**Authors:** Julio A. Lamprea‐Montealegre, Alice M. Arnold, Robyn L. McCLelland, Kenneth J. Mukamal, Luc Djousse, Mary L. Biggs, David S. Siscovick, Russell P. Tracy, Paul J. Beisswenger, Bruce M. Psaty, Joachim H. Ix, Jorge R. Kizer

**Affiliations:** ^1^ Cardiology Section San Francisco Veterans Affairs Health Care System San Francisco CA; ^2^ Kidney Health Research Collaborative San Francisco Veterans Affairs Health Care System and University of California San Francisco CA; ^3^ Department of Medicine University of California San Francisco CA; ^4^ Department of Epidemiology and Biostatistics University of California San Francisco CA; ^5^ Department of Biostatistics, School of Public Health University of Washington Seattle WA; ^6^ Cardiovascular Health Research Unit, Department of Medicine, Epidemiology and Health Services University of Washington Seattle WA; ^7^ Department of Medicine Beth Israel Deaconess Medical Center and Harvard Medical School Boston MA; ^8^ Division of Aging, Department of Medicine Brigham and Women’s Hospital and Harvard Medical School Boston MA; ^9^ The New York Academy of Medicine New York NY; ^10^ Department of Pathology and Laboratory Medicine University of Vermont College of Medicine Burlington VT; ^11^ Department of Medicine Dartmouth Geisel School of Medicine Hanover NH; ^12^ Division of Nephrology, Department of Medicine University of California San Diego CA

**Keywords:** advanced glycation endproducts, aging, cardiovascular disease, Aging, Biomarkers, Cardiovascular Disease

## Abstract

**Background:**

Advanced glycation endproducts (AGEs) have been linked to cardiovascular disease (CVD) in cohorts with and without diabetes. Data are lacking on prospective associations of various α‐dicarbonyl‐derived AGEs and incident CVD in the general population. We tested the hypothesis that major plasma AGEs are associated with new‐onset CVD in 2 population‐based cohorts of differing age and comorbidities.

**Methods and Results:**

Analyses involved a random subcohort (n=466) from the Cardiovascular Health Study and a case‐cohort sample (n=1631) from the Multi‐Ethnic Study of Atherosclerosis. Five AGEs and 2 oxidative products were measured by liquid chromatography tandem mass spectrometry. Associations with CVD (myocardial infarction and stroke) were evaluated with Cox regression. Participants in the Cardiovascular Health Study were older than the Multi‐Ethnic Study of Atherosclerosis, and had more comorbidities, along with higher levels of all AGEs. During median follow‐up of 11 years, 439 participants in the Multi‐Ethnic Study of Atherosclerosis and 200 in the Cardiovascular Health Study developed CVD. After multivariable adjustment, carboxymethyl‐lysine, 3‐deoxyglucosone hydroimidazolones and a summary variable of all measured AGEs (principal component 1) were significantly associated with incident CVD in the Cardiovascular Health Study (HRs [95% CI]: 1.20 [1.01, 1.42], 1.45 [1.23, 1.72], and 1.29 [1.06, 1.56], respectively), but not the Multi‐Ethnic Study of Atherosclerosis. Oxidative products were not associated with CVD in either cohort.

**Conclusions:**

We found α‐dicarbonyl‐derived AGEs to be associated with CVD in an older cohort, but not in a healthier middle‐aged/older cohort. Our results suggest that AGEs may exert detrimental cardiovascular effects only under conditions of marked dicarbonyl and oxidative stress. Further investigation of α‐dicarbonyl derivatives could lead to potential new strategies for CVD prevention in high‐risk older populations.

**Nonstandard Abbreviations and Acronyms**
2‐AAA: 2‐amino‐adipic acid
3DG‐H: 3‐deoxyglucosone hydroimidazolones
AGE: advanced glycation endproduct
CEL: carboxyethyl‐lysine
CHS: Cardiovascular Health Study
G‐H1: glyoxal hydroimidazolone 1
LC–MS/MS: liquid chromatography tandem mass spectrometry
MESA: Multi‐Ethnic Study of Atherosclerosis
MetSO: methionine sulfoxide
MG‐H1: methylglyoxal hydroimidazolone 1
OP: oxidative product
PC: principal component
RAGE: receptor for advanced glycation endproducts

**Clinical Perspective**

**What Is New?**

- Circulating levels of α‐dicarbonyl advanced glycation endproducts were associated with incident cardiovascular disease in a cohort with advanced age and comorbidities, but not in a healthier middle‐aged to older cohort.

**What Are the Clinical Implications?**

- These findings support the concept that α‐dicarbonyl adducts contribute to cardiovascular disease incidence only under conditions of high carbonyl or oxidative stress, as occur late in life, but this will require confirmation in populations with a broader range of age and comorbidities.
- Pending confirmation, the current results suggest that strategies to reduce formation of α‐dicarbonyls or increase their detoxification could be of benefit for cardiovascular disease prevention in people at risk.

Advanced glycation endproducts (AGEs) originate from non‐enzymatic reactions between carbonyl groups of reducing sugars and amino groups on proteins, lipids, and nucleic acids that lead to stable and profound alterations in these molecules' structure and function.^1^ Foremost among glycating species are the α‐dicarbonyls, methylglyoxal, glyoxal, and 3‐deoxyglucosone,^2^ which result from increased triose phosphate flux, degradation of glycated proteins, and oxidative stress. Such conditions supervene when cells with insulin‐independent glucose transport, such as vascular endothelial and mesenchymal cells, are subjected to glycemic excess.^3^ α‐dicarbonyls and their resulting AGE adducts have been proposed as prime molecular drivers of the vascular and renal complications of diabetes.^4^ Yet, apart from diabetes, α‐dicarbonyls and AGE adducts have also been implicated in the cardiovascular consequences of aging and chronic kidney disease (CKD), conditions characterized by heightened oxidative stress and decreased enzymatic breakdown of dicarbonyl metabolites.^5^, ^6^

Laboratory studies have shown that α‐dicarbonyls preferentially modify arginine residues enriched in the active sites of proteins, impairing functions essential for a range of cellular processes.^7^ Modification of mitochondrial electron transport chain proteins can lead to increased generation of reactive oxygen species, heightening cellular oxidative stress and promoting inflammation.^3^ α‐dicarbonyls may also access the extracellular space, where they can disturb integrin‐collagen anchoring of endothelial cells^7^; produce cross‐linking of collagen to increase vascular stiffness^5^; and modify apo‐B to enhance subendothelial LDL retention and atherogenesis.^8^ Reactive dicarbonyls can also enhance cellular expression of the receptor for AGEs (RAGE) and its ligands, promoting inflammation and oxidative stress in vascular endothelial cells.^3^ In turn, such oxidative stress, manifest through formation of protein oxidation products (OPs),^9^ can further heighten generation of AGEs, leading to a feed‐forward cycle to promote vascular disease progression.

Early clinical studies of AGEs in relation to incident cardiovascular disease (CVD) relied on skin autofluorescence as a measure of overall AGE burden or on immunoassays for circulating levels of a major AGE in human proteins, carboxymethyl‐lysine (CML). These studies found positive associations in patients with diabetes,^10^, ^11^ as well as older adults.^12^, ^13^ Subsequent use of liquid chromatography tandem mass spectrometry (LC–MS/MS) expanded these longitudinal investigations to α‐dicarbonyls, with several,^14^, ^15^, ^16^ though not all,^17^ confirming or extending the associations for plasma CML, carboxyethyl‐lysine (CEL), methylglyoxal and glyoxal with CVD events in type 1 and 2 diabetes. Assessment of the relationship of circulating α‐dicarbonyls or their derivatives with CVD in people without diabetes has been more limited. One study failed to document significant associations of these compounds with prevalent CVD,^18^ but no study to date has examined circulating α‐dicarbonyls or their AGE adducts longitudinally in relation to incident CVD in the general population.

Here we sought to extend our previous findings linking CML to incident CVD in the CHS (Cardiovascular Health Study)^13^ by leveraging LC–MS/MS measurement of α‐dicarbonyl‐derived AGEs and OPs in plasma^19^ to evaluate their associations with CVD in 2 populations: older adults from CHS, and middle‐aged to older adults free of baseline clinical CVD from the MESA (Multi‐Ethnic Study of Atherosclerosis). We tested the hypothesis that AGEs and OPs are prospectively associated with incident CVD in the setting of advanced age and substantial comorbidities, as well as among younger and healthier individuals.

## Methods

### Study Population

CHS is a prospective cohort study of CVD in older community‐dwelling adults 65 or older who were randomly selected from households identified in Medicare‐eligibility lists in 4 U.S. communities: Forsyth County, NC; Sacramento County, CA; Washington County, MD; and Allegheny County, PA.^20^ An original cohort of 5201 participants was recruited in 1989 to 1990. A supplemental cohort of 687 mostly Black participants was enrolled in 1992 to 1993. For this prospective study, from the 4082 participants with estimated glomerular filtration rate (eGFR) and available specimen at the 1992 to 1993 examination, we selected a random sample of 563 CHS participants. These participants were enriched with an additional 137 cases outside the subcohort who experienced subsequent eGFR decline for inclusion in a separate case‐cohort analysis of CKD progression. Within this total sample of 700 participants, evaluation of incident CVD was confined to the random subcohort. After excluding 97 participants with prevalent CVD from this subcohort, 466 participants were eligible for the current analysis.

MESA is a population‐based prospective cohort study of subclinical and clinical CVD in a multi‐ethnic sample aged 45 to 84.^21^ Briefly, 6814 participants without clinical CVD at baseline were recruited in 2000 to 2002 from 6 U.S. locations: New York, NY; Baltimore, MD; Chicago, IL; Los Angeles, CA; Twin Cities, MN; and Winston Salem, NC. For the current investigation, a random subcohort of 1294 participants was sampled from the 2000 to 2002 baseline visit out of a total of 5766 eligible participants with available stored plasma samples. In this subcohort, 102 participants developed a CVD event through 13 years of follow‐up. In addition, all 337 participants outside the subcohort who experienced new‐onset CVD during the same follow‐up period were selected, as were another 369 cases who developed eGFR decline for a separate aim focused on CKD progression within this ancillary study (total n=2000). The latter cases selected for the CKD progression aim were not included in the present analysis, leaving a study sample of 1631 participants with 439 incident CVD events.

Both cohort studies were approved by the institutional review boards of all participating institutions. All participants gave written informed consent before the start of the study. Requests from qualified researchers to access the data may be sent to CHS at chsdata@uw.edu or to MESA through website at https://mesa‐nhlbi.org.

### Advanced Glycation Endproducts and Oxidation Products

In CHS and MESA, plasma samples were frozen at −80°C after collection. In 2016 in CHS and 2019 in MESA, samples were thawed to send an aliquot on dry ice to PreventAGE, LLC (Lebanon, NH) for measurement. Received samples were prepared by centrifugation through 10‐K cutoff Amicon filters.^19^ This fraction contains small peptides with AGE and OP adducts, as well as free AGEs and OPs. Only the latter were selected for measurement, performed using LC–MS/MS.^19^ The primary exposures of interest were the 5 AGEs measured: methylglyoxal hydroimidazolone‐1 (MG‐H1), glyoxal hydroimidazolone‐1 (G‐H1), 3‐deoxyglucosone hydroimidazolones (3DG‐H), CML, and CEL. Secondary exposures of interest were the 2 OPs measured, methionine sulfoxide (MetSO) and 2‐aminoadipic acid (2‐AAA). Interassay coefficients of variation for the AGEs and OPs ranged from 1.5% to 5%. In MESA, 40 randomly chosen duplicates were assessed, yielding intra‐class correlation coefficients ranging from 0.96 (MetSO) to 0.99 (CML). The stability of stored AGEs and OPs over time has been demonstrated in prior analyses comparing fresh‐drawn samples with samples stored at −80°C for >10 years.^19^

### Incident Cardiovascular Disease

Incident CVD was defined as the first occurrence of fatal or non‐fatal stroke, myocardial infarction, or coronary heart disease death. Fatal coronary heart disease required a documented myocardial infarction within the previous 28 days, chest pain within the 72 hours before death, or a history of coronary heart disease, and required the absence of a known non‐atherosclerotic or non‐cardiac cause of death. In both cohorts, identification of clinical events was based on regular contacts with participants. In CHS, these involved semiannual contacts by telephone or in person,^22^ whereas in MESA, telephone contacts occurred every 9 to 12 months.^23^ Hospital records were retrieved and reviewed by an events committee for adjudication of incident CVD events using similar methods in each study.^22^, ^23^ Definite or probable myocardial infarction was defined by symptoms, ECG findings and abnormal cardiac biomarker levels. Stroke was defined as a neurologic deficit lasting ≥24 hours or associated with a lesion on brain imaging consistent with a localized ischemic or hemorrhagic stroke. In CHS, the period of follow‐up extended through 2015. For MESA, follow‐up was through 2013, at which point all incident CVD cases were selected.

### Baseline Assessments

Baseline information consisted of sociodemographic characteristics, anthropometric and blood pressure measurements, self‐reported current medications, and lifestyle behaviors. Blood chemistry measurements were performed at the same core laboratory for both cohorts,^24^ including glucose, lipid fractions, CRP (C‐reactive protein) and, in MESA, hemoglobin A1c (HbA1c) and urinary albumin/creatinine ratio. HbA1c in MESA was not available concurrently with AGE measures from Exam 1, but was measured in Exam 2 (2002–2004). Hypertension was defined as blood pressure ≥140 mm Hg (systolic), ≥90 mm Hg (diastolic) or use of anti‐hypertensive therapy. Diabetes was defined as a fasting glucose ≥126 mg/dL or use of anti‐diabetic medications including oral hypoglycemic agents or insulin. The eGFR was calculated from serum creatinine or cystatin C using the CKD Epidemiology Collaboration Equation.^25^ CKD was defined as eGFR <60 mL/min per 1.73 m^2^.

### Statistical Analysis

Descriptive statistics were used to summarize baseline demographic and clinical characteristics by incident CVD status. Comparisons applied the Kruskal‐Wallis test for continuous variables and the chi‐squared test for categorical variables. Pairwise correlations of AGEs and OPs with each other and with continuous variables were assessed by computing Spearman correlation coefficients. AGEs and OPs were modeled individually and in combination using principal component analysis. This method is efficient when analyzing a set of highly interrelated variables, as it reduces the initial number of variables to a few uncorrelated components that account for most of the variation present in all the original variables.^26^

In time‐to‐event analyses, participants were followed from the date of the baseline CHS or MESA visit to the first occurrence of a CVD event or the end of follow‐up. The associations of baseline AGEs and OPs with incident CVD events were assessed using Cox proportional hazards regression with the proportional hazard assumption tested by examining Schoenfeld residuals. Owing to the skewed distribution of AGEs and OPs, the hazard ratio (HR) estimates derived from Cox models were calculated assuming linear log–log relationships, and are presented per SD increments in the natural logarithms of the exposure measures. In MESA, risk estimates were calculated using appropriate weights for the oversampling of cases and robust variance to account for the case‐cohort design.

Sequential multivariable models were constructed to adjust for the aggregate of potential confounding as conceptualized in a directed acyclic graph (Figure S1). Models first adjusted for demographic variables including age, sex, and race or ethnicity, and then additionally for body mass index, smoking, alcohol use, diabetes, systolic blood pressure, antihypertensive medication use, LDL cholesterol, high‐density lipoprotein (HDL) cholesterol, and triglycerides. This was followed by further adjustment for CRP and eGFR, which may be both upstream and downstream of AGEs and OPs in their proposed relationship with incident CVD, and may therefore be regarded as possible confounders and at least partial mediators of this relationship. A sensitivity analysis used the cystatin C CKD Epidemiology Collaboration Equation for GFR estimation. Exploratory stratified analyses were conducted by age (stratifying by an arbitrary cut‐off of 75 years), CKD, and diabetes. To examine the possibility of non‐linear relationships, these models were compared to more flexible models that included restricted cubic splines using likelihood ratio tests. In addition, the associations of individual AGEs and OPs with incident CVD were assessed in categorical analyses using quartiles for each biomarker.

## Results

### Baseline Characteristics

Compared to the 1631 participants in MESA, the 466 CHS participants were older (median ages: 74 versus 64 years), more likely to be white (82% versus 39%) or to have CKD (30% versus 19%), with a substantially lower eGFR (68 versus 83 mL/min per 1.73 m^2^). Although median fasting glucose concentration was higher in CHS participants (98 versus 90 mg/dL), prevalence of diabetes at baseline was comparable between cohorts. In CHS and MESA, participants that developed incident CVD were older, were more likely to have glycemic dysregulation but less likely to consume alcohol and had higher blood pressure measures compared to participants that remained event free (Table 1). Proportions of women and levels of lipid measures, eGFR, and CRP were similar in participants with and without incident CVD in CHS. By contrast, participants in MESA who developed CVD were less often women, had lower HDL cholesterol and eGFR, and exhibited higher CRP compared with participants who did not.

**Table 1:** Baseline Demographic and Clinical Characteristics by Incident Cardiovascular Disease in CHS and MESA

	CHS (N=466)			MESA (N=1631)		
	No Incident CVD (n=260)	Incident CVD (n=206)	*P* value	No Incident CVD (n=1192)	Incident CVD (n=439)	*P* value
Demographic and clinical characteristics						
Age, y	73 (71–77)	74 (71–79)	0.040	62 (53–69)	69 (61–77)	<0.001
Women, n (%)	150 (58)	127 (62)	0.387	642 (54)	192 (44)	<0.001
Race or ethnicity, n (%)			0.341			0.138
White	216 (83)	167 (81)		471 (39)	176 (40)	
Black	42 (17)	39 (19)		341 (28)	131 (30)	
Chinese	NA	NA		139 (12)	34 (8)	
Hispanic	NA	NA		241 (20)	98 (22)	
BMI, kg/m^2^	26 (23–28)	25 (23–29)	0.762	28 (25–31)	28(25–32)	0.118
SBP, mm Hg	131 (120–146)	137 (126–152)	<0.001	123 (111–138)	135 (120–151)	<0.001
Antihypertensive medication, n (%)	119 (45)	102 (50)	0.421	425 (35)	244 (55)	<0.001
Hypertension, n (%)	133 (51)	121 (59)	0.103	500 (42)	306 (69)	<0.001
Diabetes, n (%)	30 (12)	37 (18)	0.048	134 (11)	105 (24)	<0.001
Fasting glucose, mg/dL	97 (91–107)	99 (92–111)	0.351	90 (83–99)	93 (85–109)	<0.001
HbA1c, %	NA	NA	NA	5.5 (5.2–5.8)	5.6 (5.3–6.1)	<0.001
Current smoker, n (%)	34 (13)	18 (9)	0.140	156 (13)	62 (14)	0.586
Current alcohol, n (%)	75 (28)	42 (20)	0.037	669 (69)	202 (57)	<0.001
LDL cholesterol, mg/dL	117 (93–140)	117 (98–141)	0.614	115 (95–138)	117 (95–135)	0.788
HDL cholesterol, mg/dL	53 (43–62)	52 (43–63)	0.508	49 (40–59)	46 (38–57)	0.002
Triglycerides, mg/dL	123 (88–161)	122 (89–168)	0.766	109 (76–160)	115 (80–166)	0.057
eGFR, mL/min per 1.73 m^2^	69 (57–80)	68 (57–80)	0.768	85 (73–96)	77 (62–90)	<0.001
Chronic kidney disease, n (%)	74 (28)	64 (31)	0.541	179 (15)	146 (33)	<0.001
UACR, mg/g	NA	NA	NA	5 (3.2–10.1)	7.8 (4.3–19.9)	<0.001
CRP, mg/L	2.3 (1.2–4.9)	2.6 (1.3–6.6)	0.083	1.9 (0.8–4.4)	2.2 (0.9–4.7)	0.091
AGE measurements, nmol/L						
CML	90 (66–117)	93 (71–122)	0.174	69 (54–93)	77 (59–104)	<0.001
3DG‐H	313 (239–416)	359 (260–471)	0.004	239 (172–330)	265 (190–385)	<0.001
CEL	67 (51–88)	71 (54–92)	0.169	52 (41–68)	58 (45–80)	<0.001
G‐H1	11.0 (8.7–13.5)	11.1 (9.3–13.7)	0.339	7.8 (6.7–9.3)	8.5 (7.2–10.3)	<0.001
MG‐H1	146 (98–239)	167 (108–256)	0.116	97 (67–152)	114 (73–190)	<0.001
OP measurements, nmol/L						
MetSO	922 (752–1166)	927 (762–1132)	0.946	698 (619–801)	714 (621–826)	0.045
2‐AAA	872 (715–1141)	863 (704–1172)	0.970	930 (736–1189)	979 (741–1245)	0.046

Cells are medians (IQR) or N (%), except as noted. 2‐AAA indicates 2‐aminoadipic acid; 3DG‐H, 3‐deoxyglucosone hydroimidazolones; AGE, advanced glycation endproduct; BMI, body mass index; CEL, carboxyethyl‐lysine; CML, carboxymethyl‐lysine; CRP, C‐reactive protein; eGFR, estimated glomerular filtration rate; G‐H1, glyoxal hydroimidazolone 1; HDL, high‐density lipoprotein; LDL, low‐density lipoprotein; MetSO, methionine sulfoxide; MG‐H1, methylglyoxal hydroimidazolone 1; NA, not available; OP, oxidation product; SBP, systolic blood pressure; and UACR, urinary albumin to creatinine ratio.

Except for 2‐AAA, whose concentrations were higher in MESA, levels of all exposure measures were substantially higher in CHS (Table 1). In MESA, concentrations of AGEs and OPs were higher among participants with than without incident CVD, but this was only the case for 3DG‐H in CHS.

### Correlations Between OPs, AGEs, and Covariates

**Figure 1:**
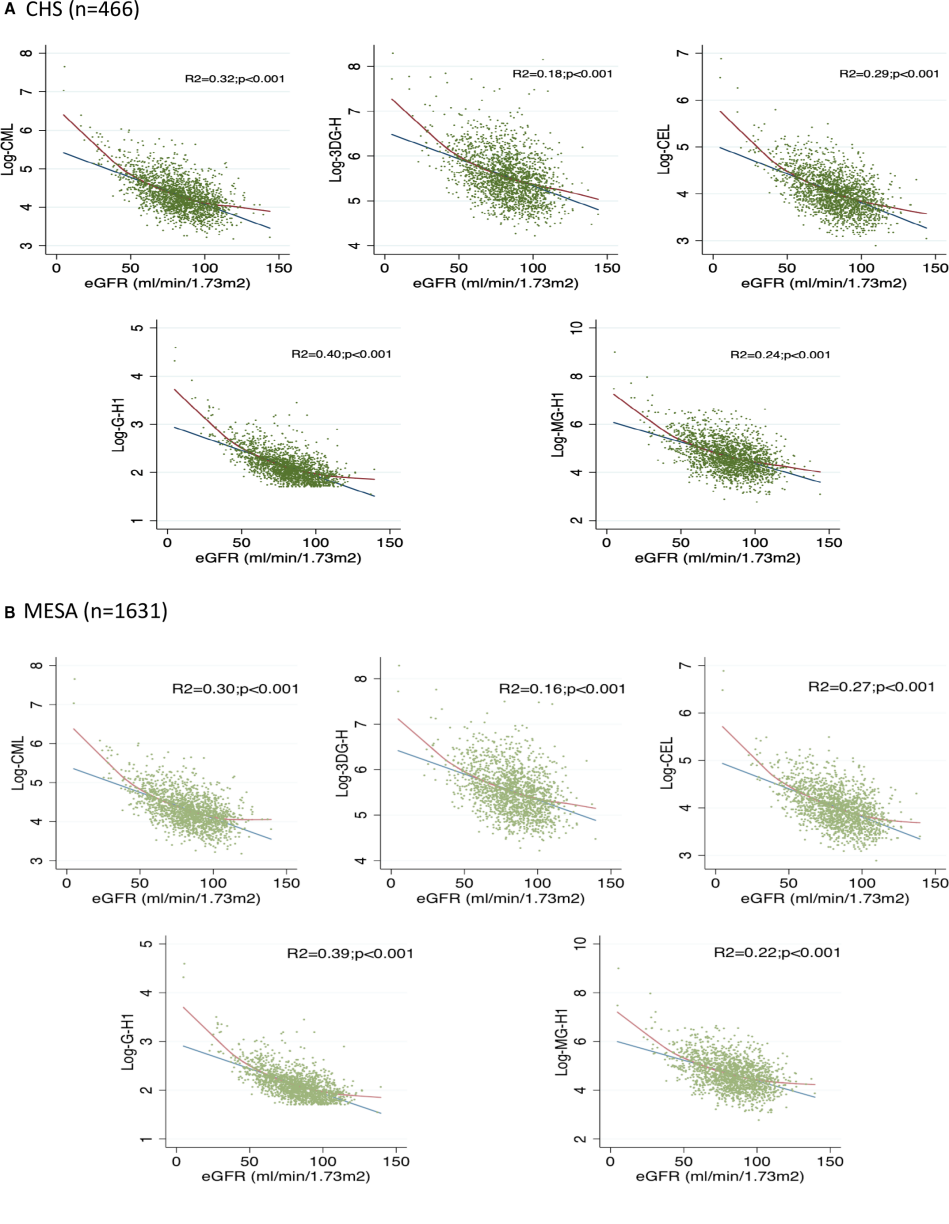
Association between estimated glomerular filtration rate and advanced glycation endproducts. Blue lines are fitted linear regression lines and red lines are fitted with a Lowess smoothing function. **A**, CHS (Cardiovascular Health Study). **B**, MESA (Multi‐Ethnic Study of Atherosclerosis). CEL indicates carboxyethyl‐lysine; CML, carboxymethyl‐lysine; 3DG‐H, 3‐deoxyglucosone hydroimidazolones; eGFR, estimated glomerular filtration rate; G‐H1, glyoxal hydroimidazolone‐1; and MG‐H1, methylglyoxal hydroimidazolone.

In CHS and MESA, there were weak correlations between OPs and between OPs and AGEs (Figure S2A and S2B). In contrast, all AGEs were strongly interrelated. Correlations were highest between CML and CEL, GH‐1 and MG‐H1, and between MG‐H1 and G‐H1 and CEL in both cohorts. Assessment of pairwise correlation coefficients of AGEs and OPs with other continuous variables showed modest or mild‐to‐moderate correlations between AGEs and age (positive), systolic blood pressure and glycemia (positive, MESA only), along with HDL cholesterol (negative). There were mild or moderate correlations of 2‐AAA with BMI, glycemia and triglycerides (positive), as well as HDL cholesterol (negative). In addition, AGEs exhibited moderate‐to‐high negative correlations with eGFR (Table S1). The proportion of the variance of each AGE that was explained by eGFR ranged from 18% with 3DG‐H and 40% with G‐H1 (Figure 1A and 1B).

The first 2 principal components (PCs) accounted for nearly 70% of the variance in all AGEs and OPs analyzed. Both PCs included the 5 AGEs and 2 OPs. PC1 accounted for the majority of the variance in AGEs with similar contributions (loadings) from all AGEs, while PC2 accounted for most of the variance in OPs (Table S2). The 2 PCs were selected as summary measures of AGEs and OPs for evaluation in Cox models of incident CVD.

### AGEs, OPs, and Risk of Cardiovascular Events

**Figure 2:**
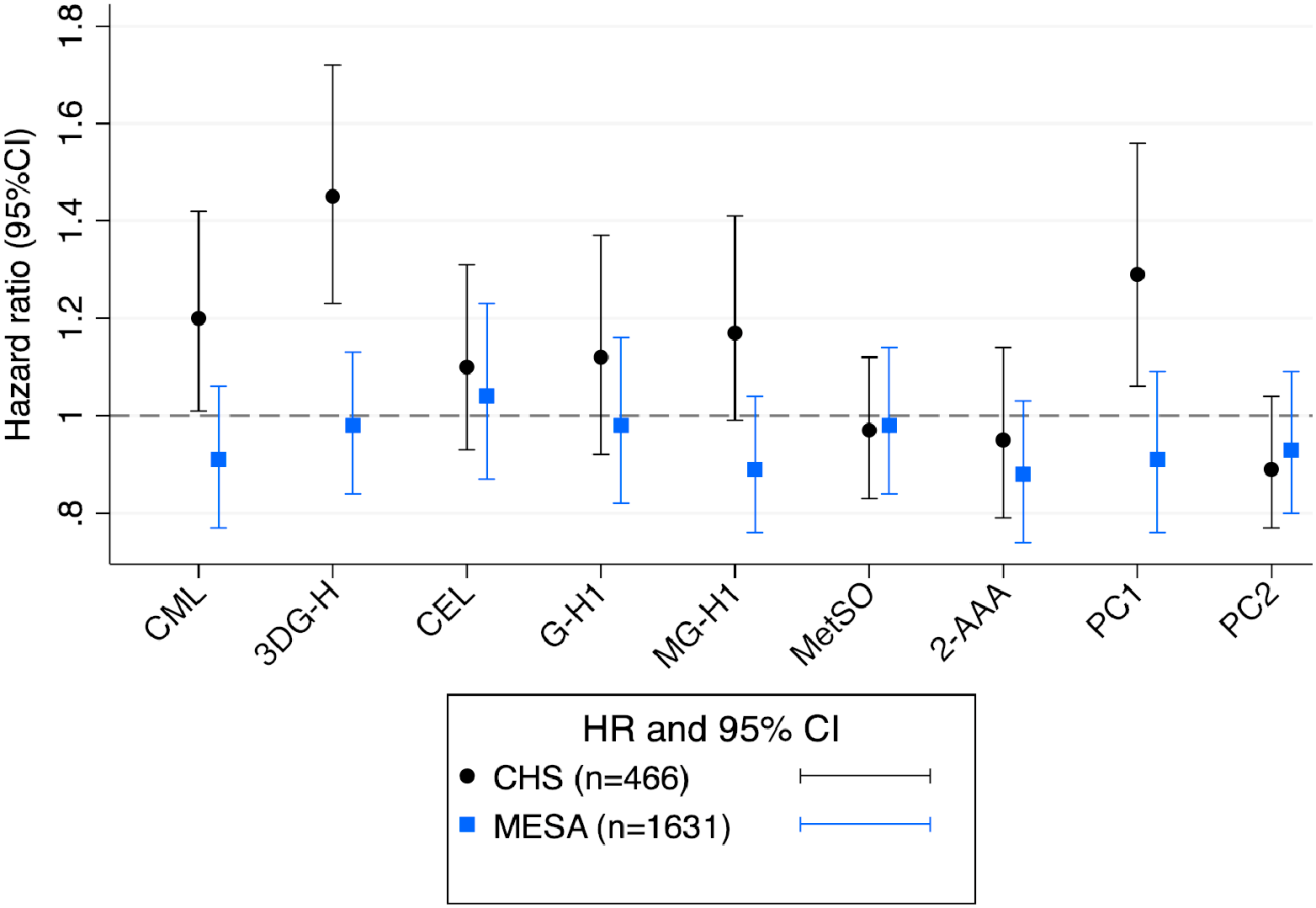
Associations of advanced glycation endproducts and oxidation products with incident cardiovascular disease in CHS (Cardiovascular Health Study) and MESA (Multi‐Ethnic Study of Atherosclerosis). Hazard ratio estimates adjusted for age, sex, race or ethnicity, body mass index, smoking, alcohol use, systolic blood pressure, use of anti‐hypertensive medication, diabetes, low‐density lipoprotein cholesterol, high‐density lipoprotein cholesterol, triglycerides, C‐reactive protein and estimated glomerular filtration rate. 2‐AAA indicates 2‐aminoadipic acid; 3DG‐H, 3‐deoxyglucosone hydroimidazolones; AGE, advanced glycation endproduct; CEL, carboxyethyl‐lysine; CML, carboxymethyl‐lysine; G‐H1, glyoxal hydroimidazolone 1; MetSO, methionine sulfoxide; MG‐H1, methylglyoxal hydroimidazolone 1; OP, oxidation product; and PC, principal component.

The maximum follow‐up time in CHS was 22 years and in MESA 13 years. During a median follow‐up of 11 years in both cohorts, 200 (42%) participants in CHS and 439 (22%) participants in MESA developed an incident CVD event. All AGEs in CHS and MESA showed significant associations with incident CVD in unadjusted models (Tables 2 and 3). In multivariable analyses, the associations of AGEs with incident CVD were attenuated after adjustment for demographic factors (Model 1), and particularly age, in both cohorts (Tables 2 and 3). In CHS, the relationships for several of the AGEs showed some additional attenuation after adjustment for lifestyle and clinical risk factors (Model 2). Upon adjustment for CRP and then eGFR (Models 3 and 4), risk estimates tended to strengthen, the positive associations for CML and 3DG‐H meeting statistical significance in these more highly adjusted models. After full adjustment including these factors (Model 4), which might act not just as confounders but also potentially as causal intermediates, each SD increment in log‐transformed CML and 3DG‐H was associated with a 20% (1% to 42%) and 45% (23% to 72%) higher risk of incident CVD, respectively (Table 2). In addition, PC1, which summarized the total variance of AGEs, was independently associated with incident CVD in CHS, with each SD increment showing a 29% (6% to 56%) higher risk of the outcome. By contrast, in MESA, nearly all associations between AGEs and incident CVD were rendered non‐significant after adjustment for demographic factors. The 2 exceptions were CEL and G‐H1, which became non‐significant after further adjustment for lifestyle and clinical risk factors in Model 2. There was no independent association for any AGE or PC1 after full adjustment including CRP and eGFR in Model 4. In turn, OPs were not significantly associated with incident CVD in crude or multivariable‐adjusted models in either CHS or MESA (Tables 2 and 3). The comparative associations in CHS and MESA of AGEs and OPs with CVD after adjustment for Model 4 covariates are displayed in Figure 2.

**Table 2:** Associations of Advanced Glycation Endproducts and Oxidation Products with Incident Cardiovascular Disease in CHS (N=466)

Continuous biomarkers	Crude		Model 1		Model 2		Model 3		Model 4	
	HR* (95% CI)	*P* value	HR* (95% CI)	*P* value	HR* (95% CI)	*P* value	HR* (95% CI)	*P* value	HR* (95% CI)	*P* value
Log CML	1.30 (1.13, 1.50)	<0.001	1.18 (1.01, 1.37)	0.029	1.13 (0.97, 1.31)	0.113	1.18 (1.01, 1.36)	0.031	1.20 (1.01, 1.42)	0.032
Log 3DG‐H	1.47 (1.28, 1.69)	<0.001	1.35 (1.17, 1.57)	<0.001	1.34 (1.15, 1.56)	<0.001	1.38 (1.19, 1.61)	<0.001	1.45 (1.23, 1.72)	<0.001
Log CEL	1.23 (1.07, 1.41)	0.003	1.12 (0.97, 1.30)	0.115	1.07 (0.92, 1.24)	0.378	1.11 (0.95, 1.28)	0.179	1.10 (0.93, 1.31)	0.251
Log G‐H1	1.30 (1.12, 1.51)	0.001	1.11 (0.94 1.30)	0.216	1.09 (0.93, 1.28)	0.300	1.11 (0.95, 1.31)	0.193	1.12 (0.92, 1.37)	0.246
Log MG‐H1	1.29 (1.11, 1.50)	0.001	1.16 (1.00, 1.35)	0.060	1.11 (0.95, 1.30)	0.196	1.16 (1.00, 1.36)	0.056	1.17 (0.99, 1.41)	0.066
Log MetSO	0.98 (0.85, 1.11)	0.728	0.97 (0.85, 1.12)	0.754	0.95 (0.82, 1.10)	0.496	0.97 (0.83, 1.12)	0.688	0.97 (0.83, 1.12)	0.677
Log 2‐AAA	1.01 (0.88, 1.16)	0.893	1.03 (0.89, 1.20)	0.649	0.98 (0.82, 1.17)	0.840	0.97 (0.81, 1.16)	0.717	0.95 (0.79, 1.14)	0.587
PC1	1.39 (1.19, 1.62)	<0.001	1.23 (1.05, 1.45)	0.011	1.18 (1.00, 1.39)	0.054	1.22 (1.04, 1.43)	0.014	1.29 (1.06, 1.56)	0.010
PC2	0.90 (0.78, 1.04)	0.159	0.95 (0.81, 1.09)	0.443	0.90 (0.77, 1.05)	0.197	0.90 (0.77, 1.05)	0.188	0.89 (0.77, 1.04)	0.177

Model 1. Adjusted for age, sex, and race or ethnicity. Model 2. Adjusted for Model 2 plus BMI, smoking, alcohol use, systolic blood pressure, use of anti‐hypertensive medication, diabetes, LDL cholesterol, HDL cholesterol, and triglycerides. Model 3. Adjusted for Model 2 plus C‐reactive protein. Model 4. Adjusted for Model 3 plus eGFR. 2‐AAA indicates 2‐aminoadipic acid; 3DG‐H, 3‐deoxyglucosone hydroimidazolones; AGE, advanced glycation endproduct; BMI, body mass index; CEL, carboxyethyl‐lysine; CHS, Cardiovascular Health Study; CML, carboxymethyl‐lysine; CRP, C‐reactive protein; eGFR, estimated glomerular filtration rate; G‐H1, glyoxal hydroimidazolone 1; HDL, high‐density lipoprotein; HR, hazard ratio; LDL, low‐density lipoprotein; MetSO, methionine sulfoxide; MG‐H1, methylglyoxal hydroimidazolone 1; OP, oxidation product; PC, principal component; and SBP, systolic blood pressure.

* Per SD higher concentration. Values of SDs on the original scale are as follows. CML: 51 nmoL/L; 3DG‐H: 190 nmoL/L; CEL: 35 nmoL/L; G‐H1: 4.2 nmoL/L; MG‐H1: 152 nmoL/L; MetSO: 346 nmoL/L; 2‐AAA: 378 nmoL/L.

**Table 3:** Associations of Advanced Glycation Endproducts and Oxidation Products with Incident Cardiovascular Disease in MESA (N=1631)

Continuous biomarkers	Crude		Model 1		Model 2		Model 3		Model 4	
	HR* (95% CI)	*P* value	HR* (95% CI)	*P* value	HR* (95% CI)	*P* value	HR* (95% CI)	*P* value	HR* (95% CI)	*P* value
Log CML	1.30 (1.17, 1.44)	<0.001	1.11 (0.99, 1.25)	0.068	0.97 (0.84, 1.11)	0.674	0.97 (0.84, 1.11)	0.685	0.91 (0.77, 1.06)	0.245
Log 3DG‐H	1.27 (1.14, 1.41)	<0.001	1.14 (1.01, 1.28)	0.027	1.01 (0.87, 1.15)	0.916	1.00 (0.87, 1.15)	0.894	0.98 (0.84, 1.13)	0.785
Log CEL	1.38 (1.24, 1.53)	<0.001	1.22 (1.08, 1.37)	0.001	1.06 (0.92, 1.22)	0.395	1.06 (0.92, 1.22)	0.394	1.04 (0.87, 1.23)	0.638
Log G‐H1	1.32 (1.21, 1.46)	<0.001	1.13 (1.01, 1.29)	0.035	1.03 (0.89, 1.18)	0.674	1.02 (0.88, 1.18)	0.738	0.98 (0.82, 1.16)	0.803
Log MG‐H1	1.29 (1.15, 1.44)	<0.001	1.13 (1.00, 1.27)	0.056	0.95 (0.82, 1.09)	0.456	0.94 (0.82, 1.08)	0.424	0.89 (0.76, 1.04)	0.152
Log MetSO	1.11 (1.00, 1.25)	0.046	1.07 (0.95, 1.21)	0.232	0.99 (0.86, 1.15)	0.962	0.99 (0.85, 1.14)	0.934	0.98 (0.84, 1.14)	0.831
Log 2‐AAA	1.11 (0.99, 1.23)	0.065	1.01 (0.89, 1.15)	0.843	0.88 (0.75, 1.04)	0.142	0.88 (0.75, 1.04)	0.146	0.88 (0.74, 1.03)	0.131
PC1	1.36 (1.22, 1.51)	<0.001	1.16 (1.02, 1.32)	0.016	0.97 (0.83, 1.13)	0.732	0.97 (0.83, 1.13)	0.732	0.91 (0.76, 1.09)	0.327
PC2	1.03 (0.93, 1.15)	0.545	1.01 (0.89, 1.14)	0.896	0.93 (0.79, 1.08)	0.371	0.93 (0.79, 1.08)	0.371	0.93 (0.80, 1.09)	0.425

Model 1. Adjusted for age, sex, and race or ethnicity. Model 2. Adjusted for Model 2 plus BMI, smoking, alcohol use, systolic blood pressure, use of anti‐hypertensive medication, diabetes, LDL cholesterol, HDL cholesterol, and triglycerides. Model 3. Adjusted for Model 2 plus C‐reactive protein. Model 4. Adjusted for Model 3 plus eGFR. 2‐AAA indicates 2‐aminoadipic acid; 3DG‐H, 3‐deoxyglucosone hydroimidazolones; AGE, advanced glycation endproduct; BMI, body mass index; CEL, carboxyethyl‐lysine; CML, carboxymethyl‐lysine; CRP, C‐reactive protein; eGFR, estimated glomerular filtration rate; G‐H1, glyoxal hydroimidazolone‐1; HDL, high‐density lipoprotein; HR, hazard ratio; LDL, low‐density lipoprotein; MESA, Multi‐Ethnic Study of Atherosclerosis; MetSO, methionine sulfoxide; MG‐H1, methylglyoxal hydroimidazolone; OP, oxidation product; PC, principal component; and SBP, systolic blood pressure.

* Per SD higher concentration. Values of SDs on the original scale are as follows. CML: 72 nmoL/L; 3DG‐H: 212 nmoL/L; CEL: 38 nmoL/L; G‐H1: 4.1 nmoL/L; MG‐H1: 240 nmoL/L; MetSO: 176 nmoL/L; 2‐AAA: 377 nmoL/L.

Sensitivity analyses that used a cystatin C formula to estimate GFR were consistent with the main findings (Tables S3 and S4 and Figure S3). In addition, in exploratory stratified analyses, the association of AGEs or OPs with CVD in CHS appeared similar when stratifying by age, CKD, or diabetes (Table S5A through S5C). There was no evidence of non‐linear associations assessed by likelihood ratio tests comparing linear models with restricted cubic spine models. Analyses of biomarker quartiles were consistent with the main findings (Table S6).

## Discussion

### Main Findings

In this study of 2 population‐based prospective cohorts, we found significant independent associations between circulating AGEs and higher incidence of CVD in one of the cohorts. The associations were observed for CML, 3DG‐H and a summary variable of all AGEs (PC1) in CHS, but not MESA, after adjustment for an array of possible confounders and factors that could act as both confounders and potentially also as causal intermediates. There was no evidence of association between OPs and incident CVD events in either cohort.

### Previous Studies

Formation of AGE adducts on proteins, nucleic acids, and lipids is a well‐recognized consequence of hyperglycemia, oxidative stress and uremia.^1^ Such AGE adducts have long been considered potential mediators of the high cardiovascular risk that accompanies diabetes, aging, and CKD.^1^ Refinement of LC–MS/MS methods to quantify major circulating AGEs and their precursors has allowed research in this area to overcome limitations of earlier techniques.^27^ Application of LC–MS/MS to cohorts with type 1 and 2 diabetes has permitted direct plasma measurement of reactive dicarbonyls, which have 20 000‐fold the glycating potential of glucose,^28^ and their resultant AGEs. Such studies have linked plasma methylglyoxal and glyoxal or a composite score of CML, CEL and pentosidine to risk of incident CVD in these contexts.^16^ Similarly, circulating MG‐H1, CEL, and CML measured with the same LC–MS/MS technique used here have been related to progression of CKD in type 1 and/or type 2 diabetes,^19^, ^29^ although an independent association with incident CVD was not documented in a sample with type 2 diabetes.^17^

To our knowledge, the present study is the first to evaluate LC–MS/MS measures of plasma AGEs, along with OPs, in relation to new‐onset CVD in a general population setting. Consistent with prior studies of older adults that employed an immunoassay to quantify circulating levels,^12^, ^13^ CML measured by LC–MS/MS was significantly associated with incident CVD in CHS. Our study newly shows that 3DG‐H was also significantly associated with higher incidence of CVD, with the numerically largest risk estimate in the same cohort. Still, all α‐dicarbonyl AGEs in CHS showed a positive risk estimate, and a summary measure was likewise significantly associated with the outcome. The lack of association documented here for any AGE in MESA, and similarly for the 2 OPs in either cohort, is also notable, and contributes important new information to the field.

### Potential Explanations

That positive associations for AGEs were observed in CHS, but not MESA, likely reflects the different characteristics of the 2 cohorts' participants. Unlike MESA participants, who were middle‐aged and older (median 64 years, range 45–84), more ethnically diverse, and free of clinically overt CVD, CHS participants were of more advanced age (median 74 years, range 65–100), mostly White and Black, and had substantial comorbidities. Nearly one‐third of our CHS sample had CKD, and over half had hypertension. Among CHS participants who did not experience incident CVD, these prevalences were approximately two‐fold and one‐quarter higher, respectively, than in their MESA counterparts. Further, although we excluded prevalent clinical CVD from our CHS sample, the CHS cohort is known to have a high prevalence of subclinical CVD.^30^ Accordingly, median concentrations of AGEs were 15% to 40% higher in CHS than MESA, and comparable to those in previous cohorts with type 1 or 2 diabetes.^19^, ^29^

The high concentrations of circulating AGEs in CHS attest to a higher level of AGE generation or reduced clearance in this cohort than in the healthier, younger adults from MESA. The reactive dicarbonyl methylglyoxal, which leads to formation of MG‐H1 and CEL, is generated from increased glycolytic flux, but also from diminished capacity of the pentose phosphate pathway to reduce triose phosphates.^1^ Glyoxal arises from fragmentation of glycated proteins and lipid peroxidation, resulting in G‐H1 and CML, while 3‐deoxyglucosone originates from enzymatic repair of fructose‐lysine residues on proteins.^1^ All 3 reactive dicarbonyls are degraded by enzymes—glyoxalase 1, aldo‐keto reductases and/or aldehyde dehydrogenases—that are impaired or downregulated in conditions of oxidative stress and inflammation.^31^ Although all measured AGEs can be intestinally absorbed as free AGE adducts, the contributions of dietary sources to their circulating levels is considered minor.^31^

As previously documented,^27^ the most abundant AGE adducts in circulation were 3DG‐H, MG‐H1, and CML. All 3 were significantly or near‐significantly associated with incident CVD in CHS. Despite these and other AGE adducts' strong negative correlations with eGFR, adjustment for the latter did not attenuate the observed associations. Nor did AGE adducts show meaningful correlations with CVD risk factors or the inflammatory marker CRP, with the exception of modest significant correlations with HDL cholesterol. Accordingly, further adjustment for such risk factors did not have a major influence on the strength of associations. This likely reflects the play of chance in our randomly selected CHS sample of moderate size, but would explain the lack of attenuation therein. More generally, the limited impact of CVD risk factors on the associations suggests that heightened generation or reduced detoxification of these AGE adducts occurs through aging‐related oxidative and non‐oxidative mechanisms that were substantially not captured by these measures.^32^

Amid suggestive or actual associations for all individual AGEs in CHS, and the significant relationship seen for their summary measure (PC1), 3DG‐H showed the strongest numerical relationship with incident CVD. The precursor of 3DG‐H, 3‐deoxyglucosone, arises primarily from non‐oxidative pathways,^32^ but its degradation by aldo‐keto reductases is diminished by oxidative stress.^31^ The pre‐eminent association observed for 3DG‐H suggests that this AGE adduct may best capture the mechanisms leading to increased carbonyl stress and adverse cardiovascular outcomes in older adults. Indeed, 3DG‐H may be an optimal measure of arginine residue modification at functionally important sites (“hotspots”) in the proteome.^1^ Among these, 3‐deoxyglucosone's demonstrated effects on histone proteins^33^ may be especially consequential through transcriptional regulation of gene expression related to atherogenesis and atherothrombosis. In turn, CML showed the second strongest and only other individually significant association with incident CVD in CHS. Although CML can be generated from glyoxal,^1^ levels of G‐H1 were not significantly associated with CVD. This supports the view that oxidation‐driven molecular rearrangements of lipids and other glycated moieties leading to CML formation may carry greater importance for CVD pathogenesis in older adults. Thus, as the foremost oxidative AGE adduct, CML may best reflect oxidative stress or its vascular effects with advancing age.^1^

Unlike AGEs, there were no significant associations between OPs and incident CVD in CHS, as in MESA. Heightened oxidative stress is both cause and consequence of AGE generation.^20^ A proposed mechanism by which AGEs exert their vascular effects is by activating pro‐inflammatory and pro‐oxidative cellular signaling pathways.^5^ Although the oxidative modification of proteins has been shown to be a marker of the cumulative effect of oxidative stress leading to vascular damage,^5^ the 2 measured OPs were only weakly correlated with AGEs in CHS and MESA. Furthermore, only the concentration of one OP, MetSO, was higher in CHS than MESA. The current findings are discordant with the inverse relationship documented previously between MetSO and incident CVD among individuals with type 2 diabetes,^17^ suggesting that neither this oxidative protein modification nor 2‐AAA are measures of oxidative stress relevant to cardiovascular risk in the general population setting.

### Implications

The present findings support the interpretation that only at high levels of dicarbonyl and oxidative stress seen with advanced age and comorbidities are the underlying pathways leading to elevated circulating concentrations of these AGE adducts or their macromolecular disruptions sufficient to heighten the incidence of cardiovascular events. This suggests non‐linear relationships of AGE adducts with incident CVD risk in broader populations, but no evidence of departure from linearity was observed in either cohort. The extent to which the measured plasma free AGE adducts—which reflect proteolytic breakdown of intra‐ and extracellular protein AGE adducts—represent markers or mediators of CVD risk in older adults cannot be determined from the present observational study. Our analyses, however, could not directly corroborate our proposed basis for the differences between CHS and MESA, as stratified analyses by advanced age or CKD in MESA did not reveal evidence of a gradient. Whether this relates to the modest size and healthy status of even the older MESA participants is uncertain. Further research is necessary to replicate the current findings in larger samples with a range of comorbidities, and to determine the basis for their heterogeneity. But the present results, when combined with evidence from cohorts with diabetes, do suggest that approaches to diminish generation of reactive dicarbonyls or enhance their detoxification could be of value for CVD prevention in such a high‐risk population. Such a strategy has been advocated for glyoxalase 1, responsible for elimination of methylglyoxal and glyoxal, through manipulation of its regulator nuclear factor erythroid 2‐related factor 2 (nrf2*)*.^31^ Pending confirmation of the current findings, such a strategy could have traction, though the relationship documented for 3DG‐H points to improvement of aldo‐keto reductase function as a possibly more effective alternative.

### Limitations

Among our study's limitations is that only subsets of participants in MESA and CHS were studied, affecting the precision of the presented estimates. Although the upper limits of the 95% CI's indicate that moderate associations remain possible for individual AGEs such as 3DG‐H, CEL, and G‐H1 in MESA, similar assessment for the summary AGE measure (PC1) excludes a moderate association. Although the PC analyses were able to reduce much of the variance in both cohorts, these results should be interpreted with caution as they are cohort specific and not readily transportable to other populations. We did not adjust for multiple comparisons for the primary analysis of AGEs, but principal component analysis of these intercorrelated individual AGE adducts revealed consistent results. Furthermore, the stratified exploratory analyses by age, CKD and diabetes status should be interpreted in the context of the study's relatively modest sample size. In addition, although we were able to control for a large number of possible confounders, we cannot exclude the possibility that residual or unmeasured confounding could account for the observed associations. In this regard, we could not meaningfully evaluate the impact of specific anti‐diabetes therapies on the associations of interest because of small numbers, particularly in CHS. We also lacked measures of post‐prandial glucose in both cohorts, and glycated hemoglobin in CHS, and their contributions to the associations under study merit further investigation.

## Conclusions

We found that circulating free AGE adducts, but not OPs, were associated with incident CVD in a sample of older adults, but not among younger, healthier individuals. Our results suggest that AGEs may be important determinants of CVD in older individuals with a higher burden of comorbidities. Pending confirmation in separate population‐based cohorts, these findings should motivate research into strategies to decrease production of, or upregulate detoxification pathways for, reactive dicarbonyls as a novel means for CVD prevention in high‐risk elders.

## Sources of Funding

This work was supported by R01 HL094555 and K24 HL135413 from the National Heart, Lung, and Blood Institute.

CHS was supported by R01 AG053325 from the National Institute on Aging; and by contracts HHSN268201200036C, HHSN268200800007C, HHSN268201800001C, N01HC55222, N01HC85079, N01HC85080, N01HC85081, N01HC85082, N01HC85083, N01HC85086, and grants U01HL080295 and U01HL130114 from the National Heart, Lung, and BI, with additional contribution from the National Institute of Neurological Disorders and Stroke. Additional support was provided by R01AG023629 from the National Institute on Aging. A full list of principal CHS investigators and institutions can be found at CHS‐NHLBI.org.

MESA was supported by contracts HHSN268201500003I, N01‐HC‐95159, N01‐HC‐95160, N01‐HC‐95161, N01‐HC‐95162, N01‐HC‐95163, N01‐HC‐95164, N01‐HC‐95165, N01‐HC‐95166, N01‐HC‐95167, N01‐HC‐95168, N01‐HC‐95169 from the National Heart, Lung, and Blood Institute, and grants UL1‐TR‐000040, UL1‐TR‐001079, and UL1‐TR‐001420 from the National Center for Advancing Translational Sciences.

## Disclosures

J.R.K. reports stock ownership in Abbott, Bristol Myers Squibb, Johnson & Johnson, Medtronic, Merck, and Pfizer. P.J.B. is Chief Medical Officer of PreventAGE Health Care, LLC, which performed the liquid chromatography tandem mass spectrometry measurements of advanced glycation endproducts and oxidation products. There are no other conflicts to report.

## Supporting information

Tables S1–S6
Figures S1–S3
