# Supplementary material for: Plasma Levels of Advanced Glycation Endproducts and Risk of Cardiovascular Events: Findings From 2 Prospective Cohorts
Source: J Am Heart Assoc. 2022 Jul 29;11(15):e024012. doi: 10.1161/JAHA.121.024012 (PMC9375486; doi:10.1161/JAHA.121.024012)

SUPPLEMENTAL MATERIAL

Table S1. Pairwise correlations between advanced oxidation and glycation products and continuous variables.

Covariate	Cohort	CML	3DG-H	CEL	G-H1	MG-H1	MetSO	2-AAA
**Age**	**CHS**	0.31*	0.29*	0.25*	0.36*	0.30*	0.10	-0.07
	**MESA**	0.25*	0.18*	0.24*	0.36*	0.21*	0.10*	0.03
**BMI**	**CHS**	-0.07	-0.04	0.02	-0.08	-0.07	-0.08	0.30*
	**MESA**	0.10*	0.02	0.09*	0.04	0.07*	0.11*	0.17*
**SBP**	**CHS**	0.13	0.11	0.08	0.05	0.07	0.08	0.00
	**MESA**	0.13*	0.10*	0.17*	0.12*	0.14*	0.03	0.09
**Fasting glucose**	**CHS**	0.00	0.02	0.04	-0.03	-0.04	-0.07	0.35*
	**MESA**	0.08*	0.03	0.13*	0.04	0.03	0.06	0.33*
**HBA1c†**	**MESA**	0.11*	0.02	0.16*	0.05	0.07	0.09	0.27*
**LDLc**	**CHS**	-0.03	-0.07	-0.03	-0.09	-0.07	-0.11	-0.03
	**MESA**	-0.03	-0.06	-0.02	-0.03	-0.04	-0.16*	-0.05
**HDLc**	**CHS**	-0.13*	-0.15*	-0.15*	-0.20*	-0.17*	0.07	-0.45*
	**MESA**	-0.10*	-0.08	-0.12*	-0.11*	-0.08	-0.04	-0.40*
**TG**	**CHS**	0.09	0.12	0.14	0.06	0.05	-0.10	0.31*
	**MESA**	0.06	0.07	0.13	0.02	0.00	-0.02	0.29*
**CRP**	**CHS**	0.05	0.05	0.05	0.03	-0.02	-0.20*	0.19
	**MESA**	0.07	-0.01	0.08	0.06	0.05	0.05	0.02
**eGFR**	**CHS**	-0.57*	-0.48*	-0.56*	-0.65*	-0.47*	0.03	-0.13*
	**MESA**	-0.50*	-0.38*	-0.48*	-0.59*	-0.41*	-0.18*	-0.08*
**UACR**	**MESA**	0.09*	0.04	0.12*	0.11*	0.06*	0.06	0.12*

*P<0.001
†HbA1c correlation coefficients obtained in 1460 participants at MESA Exam 2.

2-AAA, 2-aminoadipic acid; BMI, body mass index; CEL, carboxyethyl-lysine; CML, carboxymethyl-lysine; CRP, C-reactive protein; DBP, diastolic blood pressure; 3DG-H, 3-deoxyglucosone hydroimidazolone; G-H1, glyoxal hdroimidazolone-1; eGFR, estimated glomerular filtration rate; HbA1c, hemoglobin A1c; HDLc, high density lipoprotein cholesterol; LDLc, low density lipoprotein cholesterol; MetSO, methionine sulfoxide; MG-H1, methylglyoxal hydroimidazolone-1; SBP, systolic blood pressure; TG, triglycerides; UACR, urine albumin creatinine ratio.

Table S2. Loadings of principal components in CHS and MESA.

AGEs and OPs	CHS (n=466)		MESA (n=1631)	
	**PC1**	**PC2**	**PC1**	**PC2**
CML	0.46	-0.01	0.45	-0.06
3DG-H	0.41	-0.14	0.39	-0.14
CEL	0.44	-0.04	0.45	-0.01
G-H1	0.43	-0.02	0.43	-0.09
MG-H1	0.45	-0.10	0.46	-0.13
MetSO	0.07	0.76	0.16	0.60
2-AAA	0.13	0.61	0.11	0.76

Cells are loadings (scores). The first two principal components account for ~70% of the total variance in the data.

Table S3. Association of advanced glycation endproducts and oxidation products with incident cardiovascular disease in CHS.

Continuous Biomarker	Crude		Model 1		Model 2		Model 3		Model 4	
	HR* (95% CI)	P-value	HR* (95% CI)	P-value	HR* (95% CI)	P-value	HR* (95% CI)	P-value	HR* (95% CI)	P-value
**Log CML**	1.30 (1.13, 1.50)	<.001	1.18 (1.01, 1.37)	0.029	1.13 (0.97, 1.31)	0.113	1.18 (1.01, 1.36)	0.031	1.19 (1.01, 1.41)	0.035
**Log 3DG-H**	1.47 (1.28, 1.69)	<.001	1.35 (1.17, 1.57)	<.001	1.34 (1.15, 1.56)	<.001	1.38 (1.19, 1.61)	<.001	1.44 (1.22, 1.70)	<.001
**Log CEL**	1.23 (1.07, 1.41)	0.003	1.12 (0.97, 1.30)	0.115	1.07 (0.92, 1.24)	0.378	1.11 (0.95, 1.28)	0.179	1.10 (0.94, 1.30)	0.238
**Log G-H1**	1.30 (1.12, 1.51)	0.001	1.11 (0.94 1.30)	0.216	1.09 (0.93, 1.28)	0.300	1.11 (0.95, 1.31)	0.193	1.11 (0.92, 1.35)	0.260
**Log MG-H1**	1.29 (1.11, 1.50)	0.001	1.16 (1.00, 1.35)	0.060	1.11 (0.95, 1.30)	0.196	1.16 (1.00, 1.36)	0.056	1.17 (0.99, 1.39)	0.071
**Log MetSO**	0.98 (0.85, 1.11)	0.728	0.97 (0.85, 1.12)	0.754	0.95 (0.82, 1.10)	0.496	0.97 (0.83, 1.12)	0.688	0.97 (0.84, 1.13)	0.725
**Log 2-AAA**	1.01 (0.88, 1.16)	0.893	1.03 (0.89, 1.20)	0.649	0.98 (0.82, 1.17)	0.840	0.97 (0.81, 1.16)	0.717	0.96 (0.80, 1.15)	0.587
**PC1**	1.39 (1.19, 1.62)	<.001	1.23 (1.05, 1.45)	0.011	1.18 (1.00, 1.39)	0.054	1.22 (1.04, 1.43)	0.014	1.27 (1.05, 1.53)	0.013
**PC2**	0.90 (0.78, 1.04)	0.159	0.95 (0.81, 1.09)	0.443	0.90 (0.77, 1.05)	0.197	0.90 (0.77, 1.05)	0.188	0.91 (0.77, 1.05)	0.219

*Per SD higher concentration.
Model 1. Adjusted for age, sex and race-ethnicity.
Model 2. Adjusted for Model 2 plus BMI, smoking, alcohol use, systolic blood pressure, use of anti-hypertensive medication, diabetes, LDL cholesterol, HDL cholesterol, and triglycerides.
Model 3. Adjusted for Model 2 plus C-reactive protein.
Model 4. Adjusted for Model 3 plus cystatin-based eGFR.

Table S4. Association of advanced glycation endproducts and oxidation products with incident cardiovascular disease in MESA.

Continuous Biomarker	Crude		Model 1		Model 2		Model 3		Model 4	
	HR* (95% CI)	P-value	HR* (95% CI)	P-value	HR* (95% CI)	P-value	HR* (95% CI)	P-value	HR * (95% CI)	P-value
**Log CML**	1.30 (1.17, 1.44)	<.001	1.11 (0.99, 1.25)	0.068	0.97 (0.84, 1.11)	0.674	0.97 (0.84, 1.11)	0.685	0.89 (0.75, 1.04)	0.140
**Log 3DG-H**	1.27 (1.14, 1.41)	<.001	1.14 (1.01, 1.28)	0.027	1.01 (0.87, 1.15)	0.916	1.00 (0.87, 1.15)	0.894	0.97 (0.83, 1.12)	0.656
**Log CEL**	1.38 (1.24, 1.53)	<.001	1.22 (1.08, 1.37)	0.001	1.06 (0.92, 1.22)	0.395	1.06 (0.92, 1.22)	0.394	1.01 (0.85, 1.19)	0.894
**Log G-H1**	1.32 (1.21, 1.46)	<.001	1.13 (1.01, 1.29)	0.035	1.03 (0.89, 1.18)	0.674	1.02 (0.88, 1.18)	0.738	0.93 (0.78, 1.11)	0.406
**Log MG-H1**	1.29 (1.15, 1.44)	<.001	1.13 (1.00, 1.27)	0.056	0.95 (0.82, 1.09)	0.456	0.94 (0.82, 1.08)	0.424	0.89 (0.76, 1.03)	0.124
**Log MetSO**	1.11 (1.00, 1.25)	0.046	1.07 (0.95, 1.21)	0.232	0.99 (0.86, 1.15)	0.962	0.99 (0.85, 1.14)	0.934	0.97 (0.83, 1.12)	0.683
**Log 2-AAA**	1.11 (0.99, 1.23)	0.065	1.01 (0.89, 1.15)	0.843	0.88 (0.75, 1.04)	0.142	0.88 (0.75, 1.04)	0.146	0.87 (0.74, 1.02)	0.093
**PC1**	1.36 (1.22, 1.51)	<.001	1.16 (1.02, 1.32)	0.016	0.97 (0.83, 1.13)	0.732	0.97 (0.83, 1.13)	0.732	0.88 (0.74, 1.06)	0.176
**PC2**	1.03 (0.93, 1.15)	0.545	1.01 (0.89, 1.14)	0.896	0.93 (0.79, 1.08)	0.371	0.93 (0.79, 1.08)	0.371	0.93 (0.80, 1.08)	0.362

*Per SD higher concentration.
Model 1. Adjusted for age, sex and race-ethnicity
Model 2. Adjusted for Model 2 plus BMI, smoking, alcohol use, systolic blood pressure, use of anti-hypertensive medication, diabetes, LDL cholesterol, HDL cholesterol, and triglycerides.
Model 3. Adjusted for Model 2 plus C-reactive protein.
Model 4. Adjusted for Model 3 plus cystatin-based eGFR.

Table S5. Stratified analyses by age (**Panel A**) and the presence of diabetes (**Panel B**) and CKD (**Panel C**) in MESA and CHS.

A. Stratified analyses by age

	**CHS (N=466)**		**MESA (N=1631)**	
**Age (N sample/N events)**	**Age≤75 (292/122)**	**Age>75 (174/84)**	**Age ≤75 (1376/315)**	**Age>75 (255/124)**
	**HR* (95% CI)**	**HR* (95% CI)**	**HR* (95% CI)**	**HR* (95% CI)**
**Log CML**	1.22 (0.99, 1.50)	1.21 (0.91, 1.61)	0.90 (0.74, 1.09)	0.78 (0.53, 1.15)
**Log 3DG-H**	1.50 (1.20, 1.87)	1.25 (0.95, 1.63)	0.91 (0.77, 1.07)	1.10 (0.76, 1.60)
**Log CEL**	1.21 (0.99, 1.48)	1.06 (0.77, 1.43)	0.96 (0.80, 1.15)	1.09 (0.65, 1.84)
**Log G-H1**	1.47 (1.12, 1.90)	0.87 (0.60, 1.23)	0.91 (0.73, 1.11)	1.06 (0.77, 1.46)
**Log MG-H1**	1.37 (1.09, 1.71)	0.98 (0.73, 1.29)	0.85 (0.71, 1.01)	0.88 (0.62, 1.24)
**Log MetSO**	1.14 (0.95, 1.37)	0.77 (0.60, 1.01)	0.99 (0.83, 1.17)	1.00 (0.75, 1.36)
**Log 2-AAA**	1.01 (0.80, 1.27)	0.87 (0.22, 1.09)	0.95 (0.79, 1.13)	0.65 (0.41, 1.01)
**PC1**	1.46 (1.15, 1.85)	1.07 (0.77, 1.49)	0.85 (0.69, 1.05)	0.91 (0.61, 1.36)
**PC2**	1.03 (0.85, 1.25)	0.73 (0.55, 0.96)	1.00 (0.84, 1.18)	0.78 (0.52, 1.16)

B. Stratified analyses by diabetes status

	**CHS (N=466)**		**MESA (N=1631)**	
**Diabetes (N sample/N events)**	**No Diabetes (395/160)**	**Diabetes (67/37)**	**No Diabetes (1392/334)**	**Diabetes (239/105)**
	**HR* (95% CI)**	**HR* (95% CI)**	**HR* (95% CI)**	**HR* (95% CI)**
**Log CML**	1.24 (1.04, 1.48)	0.84 (0.53, 1.32)	0.92 (0.77, 1.09)	1.00 (0.64, 1.56)
**Log 3DG-H**	1.49 (1.24, 1.78)	0.94 (0.54, 1.62)	1.08 (0.90, 1.28)	0.76 (0.55, 1.06)
**Log CEL**	1.19 (1.01, 1.42)	0.60 (0.37, 0.97)	1.02 (0.85, 1.24)	1.21 (0.82, 1.80)
**Log G-H1**	1.13 (0.92, 1.40)	1.13 (0.69, 1.84)	0.93 (0.76, 1.13)	1.14 (0.74, 1.75)
**Log MG-H1**	1.19 (0.99, 1.43)	0.98 (0.60, 1.62)	0.91 (0.77, 1.08)	0.92 (0.57, 1.46)
**Log MetSO**	0.95 (0.81, 1.12)	1.03 (0.67, 1.59)	1.11 (0.93, 1.33)	0.82 (0.60, 1.12)
**Log 2-AAA**	0.95 (0.77, 1.17)	0.87 (0.52, 1.45)	0.94 (0.77, 1.13)	0.74 (0.47, 1.15)
**PC1**	1.32 (1.09, 1.61)	0.81 (0.47, 1.39)	0.95 (0.77, 1.15)	0.88 (0.52, 1.48)
**PC2**	0.87 (0.72, 1.04)	0.99 (0.62, 1.55)	1.05 (0.87, 1.26)	0.75 (0.52, 1.05)

C. Stratified analyses by CKD status

	CHS (N=466)		MESA (N=1631)	
**CKD (N sample/N events)**	**No CKD (288/125)**	**CKD (178/81)**	**No CKD (1308/294)**	**CKD (323/145)**
	**HR* (95% CI)**	**HR* (95% CI)**	**HR* (95% CI)**	**HR* (95% CI)**
**Log CML**	1.21 (1.00, 1.48)	1.09 (0.82, 1.45)	0.93 (0.77, 1.11)	0.92 (0.72, 1.17)
**Log 3DG-H**	1.42 (1.16, 1.72)	1.23 (0.92, 1.64)	1.01 (0.86, 1.20)	0.91 (0.69, 1.18)
**Log CEL**	1.16 (0.95, 1.40)	0.85 (0.61, 1.18)	1.04 (0.86, 1.26)	0.99 (0.76, 1.29)
**Log G-H1**	1.11 (0.90, 1.38)	1.07 (0.77, 1.48)	0.90 (0.72, 1.11)	1.07 (0.85, 1.33)
**Log MG-H1**	1.19 (0.97, 1.44)	1.01 (0.72, 1.43)	0.92 (0.77. 1.09)	0.93 (0.72, 1.20)
**Log MetO**	0.97 (0.81, 1.17)	0.92 (0.71, 1.19)	1.05 (0.88, 1.27)	0.97 (0.74, 1.26)
**Log 2-AAA**	1.08 (0.87, 1.35)	0.70 (0.48, 1.00)	0.96 (0.79, 1.15)	0.66 (0.46, 1.00)
**PC1**	1.28 (1.03, 1.60)	1.04 (0.73, 1.46)	0.93 (0.73, 1.15)	0.93 (0.72, 1.21)
**PC2**	0.95 (0.78, 1.15)	0.80 (0.60, 1.05)	1.02 (0.85, 1.21)	0.80 (0.58, 1.11)

*Per SD higher concentration.
Hazard ratio estimates adjusted for age, sex, race/ethnicity, body mass index, smoking, alcohol use, systolic blood pressure, use of anti-hypertensive medication, diabetes, low density lipoprotein cholesterol, high density lipoprotein cholesterol, triglycerides, C-reactive protein and estimated glomerular filtration rate.

Table S6. Categorical associations of advanced glycation and oxidation end products with incident CVD.

CHS				MESA			
**Categorical predictor (quartile)**	**N at risk (N events)**	**HR (95% CI)**	**P-value**	**Categorical predictor (quartile)**	**N at risk (N events)**	**HR (95% CI)**	**P-value**
**CML**				**CML**			
≤67 nmol/l	114 (45)	Referent		≤55 nmol/l	416 (88)	Referent	
>67 - ≤90 nmol/l	114 (51)	1.09 (0.70, 1.70)	0.677	>55 - ≤71 nmol/l	409 (99)	1.13 (0.75, 1.69)	0.553
>90 - ≤117 nmol/l	120 (56)	1.27 (0.81, 2.00)	0.293	>71 - ≤97 nmol/l	407 (122)	1.02 (0.68, 1.55)	0.889
>117 nmol/l	118 (54)	1.31 (0.81, 2.10)	0.271	>97 nmol/l	399 (130)	0.82 (0.52, 1.29)	0.405
**3DG-H**				**3DG-H**			
≤246 nmol/l	116 (42)	Referent		≤176 nmol/l	407 (84)	Referent	
>246 - ≤327 nmol/l	117 (51)	1.33 (0.86, 2.07)	0.196	>176 - ≤245 nmol/l	411 (108)	1.25 (0.83, 1.87)	0.274
>327 - ≤436 nmol/l	116 (51)	1.51 (0.95, 2.37)	0.075	>245 - ≤344 nmol/l	408 (110)	1.11 (0.74, 1.68)	0.592
>436 nmol/l	117 (62)	2.28 (1.42, 3.67)	0.001	>344 nmol/l	405 (137)	1.03 (0.66, 1.60)	0.890
**CEL**				**CEL**			
≤52 nmol/l	111 (45)	Referent		≤42 nmol/l	440 (92)	Referent	
>52 - ≤69 nmol/l	119 (52)	1.01 (0.65, 1.56)	0.945	>42 - ≤54 nmol/l	390 (100)	1.08 (0.71, 1.62)	0.714
>69 - ≤90 nmol/l	119 (52)	1.18 (0.75, 1.84)	0.474	>54 - ≤72 nmol/l	399 (99)	0.74 (0.49, 1.11)	0.152
>90 nmol/l	117 (57)	1.16 (0.72, 1.86)	0.530	>72 nmol/l	402 (148)	1.09 (0.71, 1.67)	0.687
**G-H1**				**G-H1**			
≤9 nmol/l	114 (42)	Referent		≤6.8 nmol/l	365 (79)	Referent	
>9 - ≤11 nmol/l	114 (59)	1.66 (1.08, 2.55)	0.020	>6.8 - ≤8 nmol/l	432 (99)	0.98 (0.66, 1.48)	0.952
>11 - ≤14 nmol/l	119 (52)	1.21 (0.75, 1.94)	0.434	>8 - ≤9.7 nmol/l	383 (114)	0.83 (0.54, 1.28)	0.411
>14 nmol/l	119 (53)	1.37 (0.82, 2.29)	0.228	>9.7 nmol/l	451 (147)	0.89 (0.58, 1.37)	0.627
**MG-H1**				**MG-H1**			
≤100 nmol/l	115 (44)	Referent		≤67 nmol/l	411 (95)	Referent	
>100 - ≤155 nmol/l	114 (50)	1.16 (0.75, 1.79)	0.488	>67 - ≤102 nmol/l	408 (98)	0.99 (0.67, 1.46)	0.978
>155 - ≤246 nmol/l	120 (57)	1.12 (0.72, 1.74)	0.592	>102 - ≤161 nmol/l	408 (104)	0.81 (0.55, 1.21)	0.308
>246 nmol/l	117 (55)	1.35 (0.84, 1.17)	0.202	>161 nmol/l	404 (142)	0.79 (0.52, 1.19)	0.256
**MetSO**				**MetSO**			

≤759 nmol/l	116 (48)	Referent		≤619 nmol/l	408 (107)	Referent	
>759 - ≤925 nmol/l	116 (53)	1.09 (0.73, 1.64)	0.646	>619 - ≤703 nmol/l	410 (97)	0.89 (0.61, 1.31)	0.569
>925 - ≤1137 nmol/l	118 (56)	0.98 (0.64, 1.48)	0.913	>703 - ≤811 nmol/l	408 (109)	0.93 (0.61, 1.31)	0.706
1137 nmol/l	116 (49)	0.91 (0.59, 1.39)	0.681	>811 nmol/l	405 (126)	0.85 (0.56, 1.28)	0.441
**2-AAA**				**2-AAA**			
≤708 nmol/l	115 (53)	Referent		≤740 nmol/l	408 (109)	Referent	
>708 - ≤872 nmol/l	118(50)	0.83 (0.54, 1.27)	0.398	>740 - ≤947 nmol/l	409 (88)	0.67 (0.45, 1.00)	0.054
>872 - ≤1150 nmol/l	117 (49)	0.73 (0.47, 1.13)	0.163	>947 - ≤1213 nmol/l	408 (118)	0.65 (0.43, 0.99)	0.045
>1150 nmol/l	116 (54)	0.83 (0.52, 1.32)	0.444	>1213 nmol/l	406 (124)	0.77 (0.49, 1.20)	0.248

Hazard ratio estimates adjusted for age, sex, race/ethnicity, body mass index, smoking, alcohol use, systolic blood pressure, use of anti-hypertensive medication, diabetes, low density lipoprotein cholesterol, high density lipoprotein cholesterol, triglycerides, C-reactive protein and estimated glomerular filtration rate.

Figure S1. Directed acyclic graph for the association between advanced glycation products or oxidative products and CVD. T1* = Tine 1.

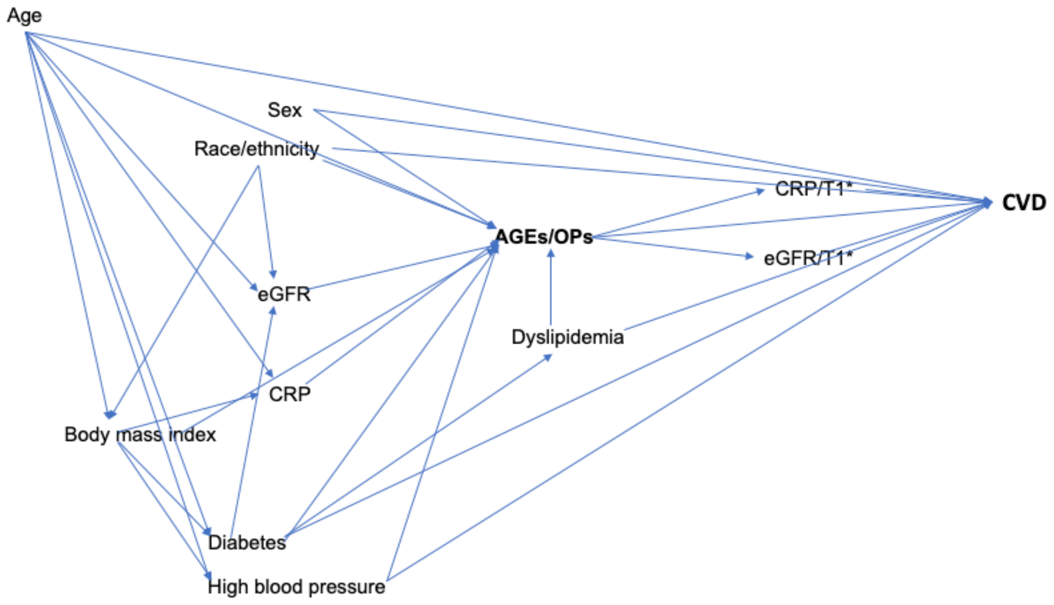

Figures S2A and S2B. Correlations between advanced oxidation and glycation products in CHS and MESA.

A. CHS (n=466)

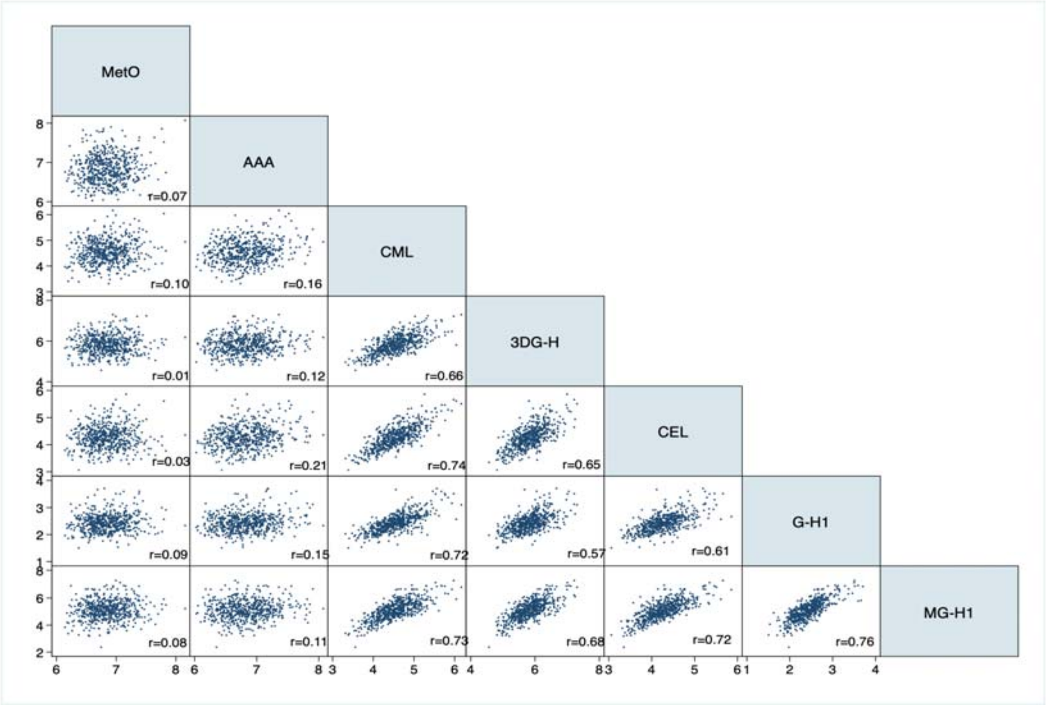

B. MESA (N=1631)

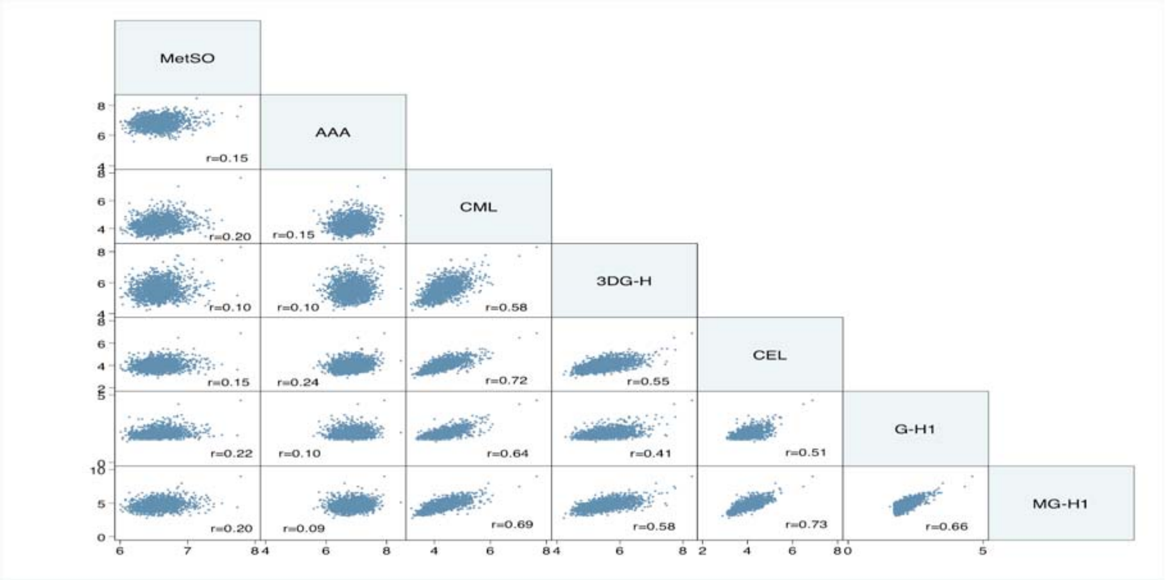

Figures S3A and S3B. Association between cystatin C estimated GFR and advanced glycation products in CHS and MESA. Continuous lines are fitted linear regression lines and dashed lines are fitted with a Lowess smoothing function.

A. CHS (N=466)

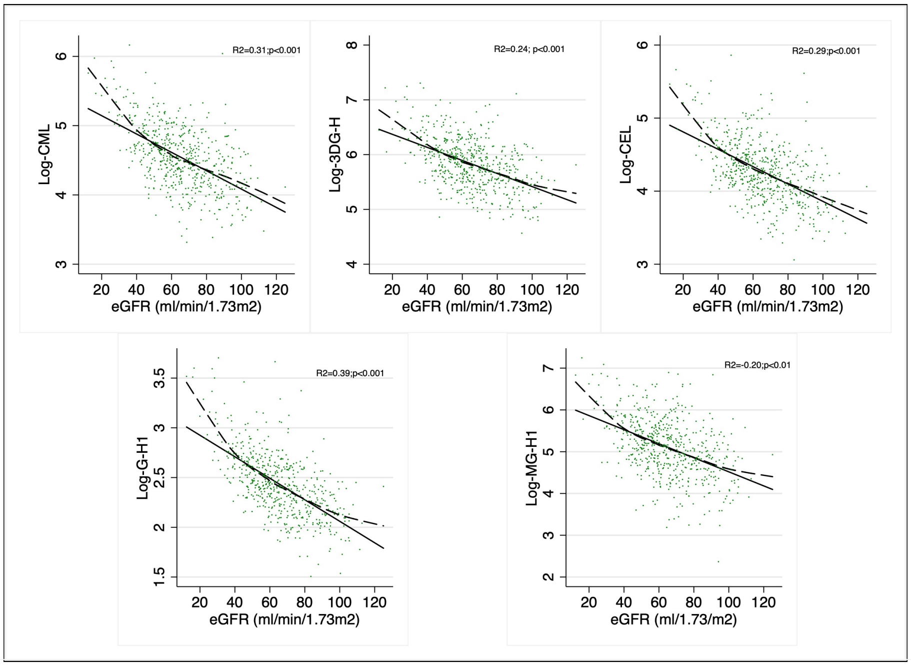

B. MESA (N=1631)

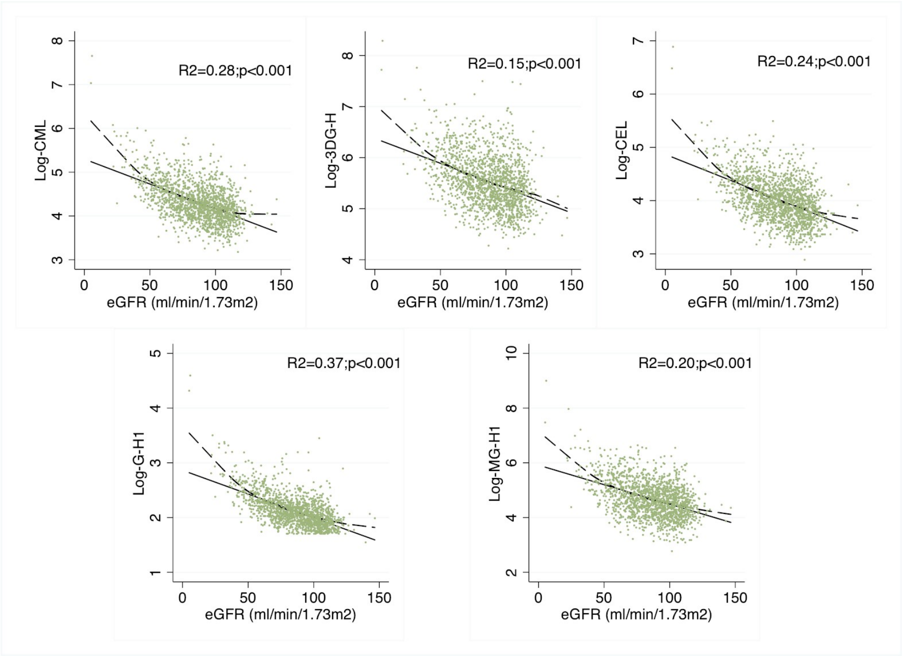

Supplement: Supplementary file 1 — Tables S1–S6 Figures S1–S3 [file JAH3-11-e024012-s001.pdf]
